# Overexpression of acylglycerol kinase is associated with poorer prognosis and lymph node metastasis in nasopharyngeal carcinoma

**DOI:** 10.1007/s13277-015-4148-x

**Published:** 2015-10-07

**Authors:** Qian Zhu, Su-Mei Cao, Huan-Xin Lin, Qi Yang, Sai-Lan Liu, Ling Guo

**Affiliations:** 1 Department of Nasopharyngeal Carcinoma, Sun Yat-sen University Cancer Center, Guangzhou, 510060 Guangdong China; 2 Department of Preventive Oncology, Sun Yat-sen University Cancer Center, Guangzhou, 510060 Guangdong China; 3 Department of Radiation Oncology, Sun Yat-sen University Cancer Center, Guangzhou, 510060 Guangdong China; 4 State Key Laboratory of Oncology in South China and Collaborative Innovation Center for Cancer Medicine, Guangzhou, China

**Keywords:** Acylglycerol kinase, Biomarker, Nasopharyngeal cancer, Lymph node metastasis, Prognosis

## Abstract

Acylglycerol kinase (AGK) has been reported to promote a malignant phenotype and enhance the development of cancer stem cells. However, the clinical value of AGK in cancer remains unclear. This study aimed to investigate the expression and clinicopathological significance of AGK in nasopharyngeal carcinoma (NPC). AGK was significantly upregulated in NPC cell lines and clinical specimens as indicated by real-time PCR and Western blotting. Among the AGK-positive cases, 52/114 (45.6 %) of the archived human NPC specimens expressed high levels of AGK. High expression of AGK was associated with significantly shorter overall and disease-free survival (*P* < 0.001 and *P* = 0.002; log-rank test) and was an independent prognostic factor for overall survival (*P* = 0.041; multivariate Cox analysis). High AGK expression was associated with lymph node metastasis (*P* < 0.001; chi-squared test) and was an independent predicted factor for lymph node metastasis in NPC (*P* = 0.032; multivariate logistic analysis). AGK is overexpressed and associated with disease progression and lymph node metastasis in NPC. AGK has potential as a novel prognostic factor for overall survival in NPC.

## Introduction

Nasopharyngeal carcinoma (NPC) is a malignant cancer arising from the epithelial surface of the nasopharynx. NPC is especially common in Southern China and Southeast Asia [1, 2], with the highest incidence in the world observed among the Cantonese-speaking population in Guangdong Province [3, 4]. As a result of technical improvements in radiotherapy delivery such as intensity-modulated radiation therapy (IMRT), excellent local control can now be achieved; however, local recurrence and metastasis remain the leading causes of mortality in advanced stage disease [5]. Previous clinical study has demonstrated that the neck nodal status is an independent prognostic factor that affects overall survival in patients with NPC without distant metastasis [6]. Involvement of both cervical lymph nodes and retropharyngeal lymph nodes has prognostic value for N1 patients with NPC [7]. Therefore, identification of molecular markers for prognosis and lymph node metastasis may help to improve the overall survival rate and design individualized therapeutic regimens in patients with NPC.

Acylglycerol kinase (AGK) has been found to be abundantly expressed in the heart, muscle, kidney, and brain [8]. By acting as a lipid kinase, AGK catalyzes the phosphorylation of acylglycerol to generate lysophosphatidic acid (LPA) [8–11], which is known to be involved in tumor progression [12], invasion, neovascularization, and metastasis [13]. AGK was reported to be overexpressed in prostate, breast, esophageal squamous cell carcinoma (ESCC), and oral squamous cell carcinoma [8, 14–17]. Bektas et al. demonstrated that overexpression of AGK enhanced the proliferation and migration of prostate cancer cells in vitro [8]. AGK expression was significantly associated with the primary tumor Gleason grade and prostatic capsular invasion in prostate cancer [14]. Wang et al. showed that AGK promotes cell proliferation and tumorigenicity in breast cancer [15]. Overexpression of AGK promoted a stem cell-like phenotype in human ESCC cells in vivo and tumorigenicity in vitro [16]. Recently, AGK is reported to promote cell proliferation and cell cycle progression in oral squamous cell carcinoma [17]. Taken together, these findings indicate that AGK may play an essential role in the progression and development of cancer. However, the expression and clinical significance of AGK in NPC remain unclear.

In the present study, we aimed to investigate the expression of AGK in NPC and explore its relationship with the clinicopathological features and prognosis of patients with NPC. We demonstrate that AGK is significantly upregulated in NPC and overexpression of AGK is closely associated with the clinical stage, T, N, M classification, histological differentiation, and lymph node metastasis. Moreover, Cox regression analysis revealed that AGK may be considered as an independent biomarker of prognosis in NPC. Multivariate logistic analysis revealed that AGK may also represent an independent biomarker for lymph node metastasis in NPC. Collectively, these findings strongly suggest that AGK plays a significant role in NPC progression and lymph node metastasis.

## Materials and methods

### Cell lines

The primary normal nasopharyngeal epithelial cell line NP69 was obtained from Dr. George SW Tsao, Cancer Center, Hong Kong University, Hong Kong, and cultured in keratinocyte/serum-free medium (Invitrogen, Grand Island, NY, USA). Nasopharyngeal cancer cell lines (CNE-1, CNE-2, SUNE-1, 6-10B, 5-8F, and HK-1) were cultured in DMEM medium (Gibco, Grand Island, NY, USA) supplemented with 10 % FBS (HyClone, Logan, UT, USA).

### Tissue specimens and patient information

NPC paraffin-embedded specimens from a total of 114 patients who had been histopathologically and clinically diagnosed with NPC at the Cancer Center of Sun Yat-sen University (Guangzhou, China) between 2007 and 2010 were used in the present study. Prior patient consent and approval from Sun Yat-sen University Cancer Center Institutional Review Board were obtained. Tumor grade and stage were defined according to the seventh edition of the UICC staging system. One patient (0.9 %) had stage I disease, 14 (12.3 %) had stage II, 48 (42.1 %) had stage III, and 51 (44.7 %) had stage IV. All of the NPC patients received radiotherapy or concurrent chemoradiotherapy, while distant metastasis NPC patients underwent a long period of induction chemotherapy to control the metastatic lesions. The clinicopathological features of the patients are summarized in Table 1. The follow-up time for the primary NPC cohort ranged from 17 to 77 months, and the median follow-up time was 60 months. The percentage of tumor purity in sections adjacent to the regions used for RNA extraction was estimated during routine histopathological analysis.

**Table 1:** Association between AGK expression and the clinicopathological features of nasopharyngeal cancer

Feature	No. of patients	AGK expression		*P* value
		Low	High	
Gender				
Male	82 (71.9 %)	45 (54.9 %)	37 (45.1 %)	0.866
Female	32 (28.1 %)	17 (53.1 %)	15 (46.9 %)	
Age (years)				
≤45	65 (57.0 %)	38 (58.5 %)	27 (41.5 %)	0.314
>45	49 (43.0 %)	24 (50.0 %)	25 (50.0 %)	
T classification				
T1	1 (0.90 %)	1 (100 %)	0 (0.0 %)	0.012
T2	19 (16.7 %)	16 (84.2 %)	3 (15.8 %)	
T3	54 (47.4 %)	29 (53.7 %)	25 (46.3 %)	
T4	40 (35.1 %)	16 (40.0 %)	24 (60.0 %)	
N classification				
N0	19 (16.7 %)	18 (94.7 %)	1 (5.3 %)	<0.001
N1	49 (43.0 %)	32 (65.3 %)	17 (34.7 %)	
N2	33 (28.9 %)	10 (30.3 %)	23 (69.7 %)	
N3	13 (11.4 %)	2 (15.4 %)	11 (84.6 %)	
M classification				
M0	108 (94.7 %)	62 (54.7 %)	46 (42.6 %)	0.006
M1	6 (5.3 %)	2 (33.3 %)	4 (66. 7 %)	
Clinical stage				
I	1 (0.90 %)	1 (100.0 %)	0 (0.0 %)	<0.001
II	14 (12.3 %)	10 (71.4 %)	4 (28.6 %)	
III	48 (42.1 %)	29 (60.4 %)	19 (39.6 %)	
IV	51 (44.7 %)	18 (35.3 %)	33 (64.7 %)	
IV classification				
IVa	35 (68.6 %)	14 (40.0 %)	21 (60.0 %)	
IVb	10 (19.6 %)	2 (20.0 %)	8 (80.0 %)	0.080
IVc	6 (11.8 %)	2 (33.3 %)	4 (66.7 %)	
Histological differentiation				
U	85 (74.6 %)	36 (42.4 %)	49 (57.6 %)	
D	29 (25.4 %)	26 (89.7 %)	3 (10.3 %)	<0.001
Lymph node metastasis				
Yes	95 (83.3 %)	44 (46.3 %)	51 (53.7 %)	<0.001
No	19 (16.7 %)	18 (94.7 %)	1 (5.3 %)	
Vital status				
Alive	96 (84.2 %)	61 (63.5 %)	35 (36.5 %)	<0.001
Dead	18 (15.8 %)	1 (5.6 %)	17 (94.4 %)	

*D* differentiated nonkeratinized carcinoma, *U* undifferentiated nonkeratinized carcinoma

The freshly frozen NPC tissues and noncancerous nasopharyngeal tissue were obtained, after informed consent, from the patients who underwent nasopharyngeal biopsy before treatment. Two paired tumor samples and the adjacent noncancerous tissues (N1–2) were from the same patients, while five noncancerous tissues (T3–7) were from the additional tumor samples. All the tissues were pathologically diagnosed in Cancer Center of Sun Yat-sen University.

### Real-time PCR

Total RNA was extracted from the cell lines and freshly frozen tissues using TRIzol reagent (Invitrogen, Carlsbad, CA, USA) according to the manufacturer’s instructions, treated with RNase-free DNase, and 2 μg was used for complementary DNA (cDNA) synthesis with random hexamers. For PCR amplification of *AGK* cDNA using *AGK*-specific primers, initial amplification was performed with denaturation at 95 °C for 10 min, followed by 28 cycles of denaturation at 95 °C for 60 s, primer annealing at 58 °C for 30 s, and primer extension at 72 °C for 30 s. Upon completion of the cycling steps, a final extension step was performed at 72 °C for 5 min; then, the reaction mixtures were stored at 4 °C. Real-time PCR was performed to measure the relative fold change in AGK messenger RNA (mRNA) expression in each of the primary tumor specimens relative to the normal nasopharyngeal tissues (collected from different patients). The primers and probe were designed using Primer Express v2.0 (Applied Biosystems), and the sequences were as follows: AGK forward, 5′-CGAAGGCTTGCGTCCTACTG-3′ and reverse, 5′-TGGTGGACAGCTGCACATCT-3′ and glyceraldehyde-3-phosphate dehydrogenase (GAPDH) forward 5′-AAGGTCATCCCTGAGCTGAA-3′ and reverse 5′-TGACAAAGTGGTCGTTGAGG-3′. Expression data were normalized to the geometric mean of *GAPDH* and calculated as 2^−[(Ct of AGK) − (Ct of GAPDH)]^, where Ct represents the threshold cycle for each transcript; all experiments were performed in triplicate.

### Western blotting

Cells at 70 to 80 % confluence were washed twice with ice-cold phosphate-buffered saline (PBS) and lysed on ice in radioimmunoprecipitation assay buffer (RIPA; Cell Signaling Technology, Danvers, MA, USA) containing complete protease inhibitor cocktail (Roche Applied Sciences, Mannheim, Germany), then heated for 5 min at 100 °C. Freshly human tissue samples were ground to a powder in liquid nitrogen and lysed in sodium dodecyl sulfate (SDS)-PAGE sample buffer. Equal amounts of protein (20 μg) were separated on 10.5 % SDS polyacrylamide gels and transferred to PVDF membranes (Immobilon P; Millipore, Bedford, MA, USA). The membranes were blocked with 5 % fat-free milk in Tris-buffered saline containing 0.1 % Tween-20 (TBST) for 1 h at room temperature, incubated with anti-AGK-2 antibody (1:1000, ab96507; Abcam, USA) overnight at 4 °C. α-Tubulin mouse monoclonal antibody (1:1000, Sigma, St. Louis, MO, USA) was used as an internal loading control. Protein bands were detected using ECL prime Western blotting detection reagent (Amersham Biosciences Europe, Freiberg, Germany) according to the manufacturer’s instructions.

### Immunohistochemical analysis

Briefly, 4-μm-thick paraffin sections were deparaffinized in xylene, rehydrated, microwaved in EDTA antigen retrieval buffer, treated with 3 % hydrogen peroxide in methanol to quench endogenous peroxidase activity, incubated with 1 % bovine serum albumin to block nonspecific binding, and then incubated with anti-AGK-2 rabbit polyclonal antibody (1:100; ab96507; Abcam) overnight at 4 °C. Normal goat serum was used as a negative control. After washing, the tissue sections were incubated with a biotinylated anti-rabbit secondary antibody (Abcam), followed by streptavidin-horseradish peroxidase complex (Abcam), developed using 3-amino-9-ethyl carbazole, counterstained with 10 % Mayer’s hematoxylin, dehydrated, and mounted in Crystal Mount (Company). Twenty cases were used for normal controls. The percentage of positively stained tumor cells was scored as 1 (<25 % positive tumor cells), 2 (25–50 %), 3 (50–75 %), or 4 (>75 %). The staining intensity was graded as 0 (no staining), 1 (weak staining, light yellow), 2 (moderate staining, yellow brown), or 3 (strong staining, brown). The overall staining score was determined by multiplying the score for the percentage of positively stained tumor cells by the score for the staining intensity (the possible scores were 0, 1, 2, 3, 4, 6, 8, 9, and 12), and the scores determined by two independent investigators were averaged for each sample. The cutoff value for AGK was chosen on the basis of a measure of heterogeneity using the log-rank test with respect to overall survival (OS); a score of ≥9 was used to define tumors with high AGK expression and <9 with low AGK expression.

Immunohistochemical staining for protein expression in tumor and normal tissues was quantitatively analyzed with the AxioVision Rel.4.6 computerized image analysis system assisted with the automatic measurement program (Carl Zeiss). Briefly, the stained sections were evaluated at 200 magnification, and 10 representative staining fields of each section were analyzed to verify the mean absorbance, which represents the strength of staining signals as measured per positive pixels. The mean absorbance data were statistically analyzed using *t* test to compare the average mean absorbance difference between different groups of tissues, and *P* < 0.05 was considered significant.

### Statistical analysis

All statistical analyses were performed using SPSS 16.0. The chi-squared test was used to investigate the relationship between AGK expression and the clinicopathologic features of NPC. Bivariate correlations between study variables were evaluated using the Spearman’s rank correlation analysis. Survival curves were plotted using the Kaplan-Meier method and compared using the log-rank test. Clinicopathological characteristics that are extensively used to predict prognosis in clinical practice and AGK expression were evaluated using univariate analysis and in multivariate Cox regression analyses using a Cox proportional hazards model with forward selection. Multivariate logistic regression analysis was performed to identify the predictive factor for lymph node metastasis in NPC.

In all analyses, *P* values <0.05 were considered statistically significant.

## Results

### AGK is overexpressed in NPC cell lines and human NPC tissues

**Fig. 1:**
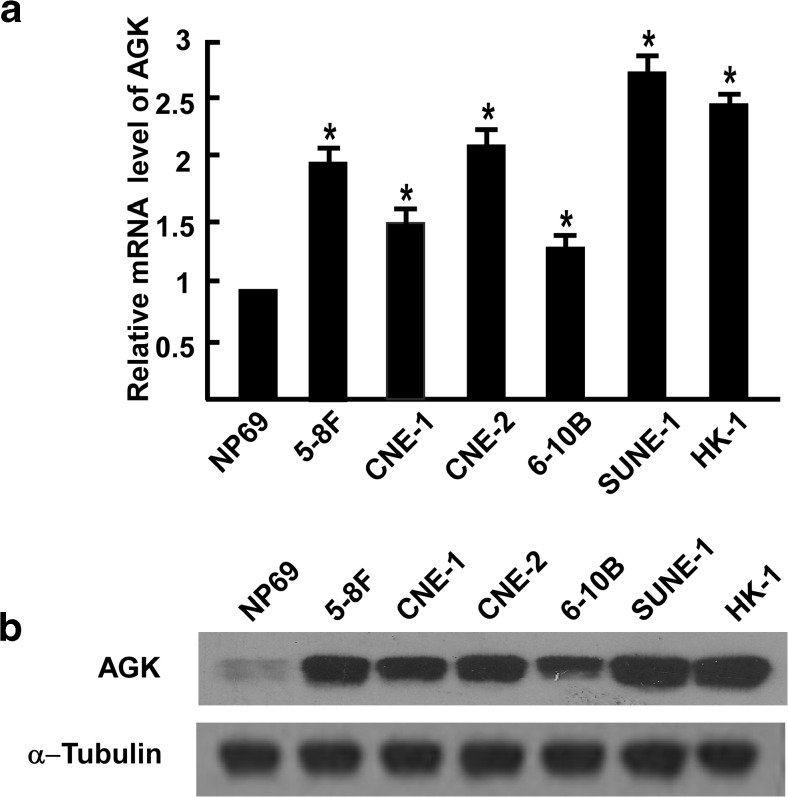
Real-time PCR (**a**) and Western blotting (**b**) analysis of AGK mRNA and protein expression in a normal nasopharyngeal cell line (NP69) and six nasopharyngeal cancer cell lines (5-8F, CNE-1, CNE-2, 6-10B, SUNE-1, and HK-1). *Error bars* are standard deviation of the mean (SD) calculated from three experiments performed in parallel

*AGK* mRNA was expressed at higher levels in all six NPC cell lines tested than the normal nasopharyngeal epithelial line NP69 (Fig. 1a). Similarly, high levels of AGK protein expression were observed in the NPC cell lines whereas only low levels of AGK were detected in NP69 primary normal nasopharyngeal epithelial cells (Fig. 1b).

**Fig. 2:**
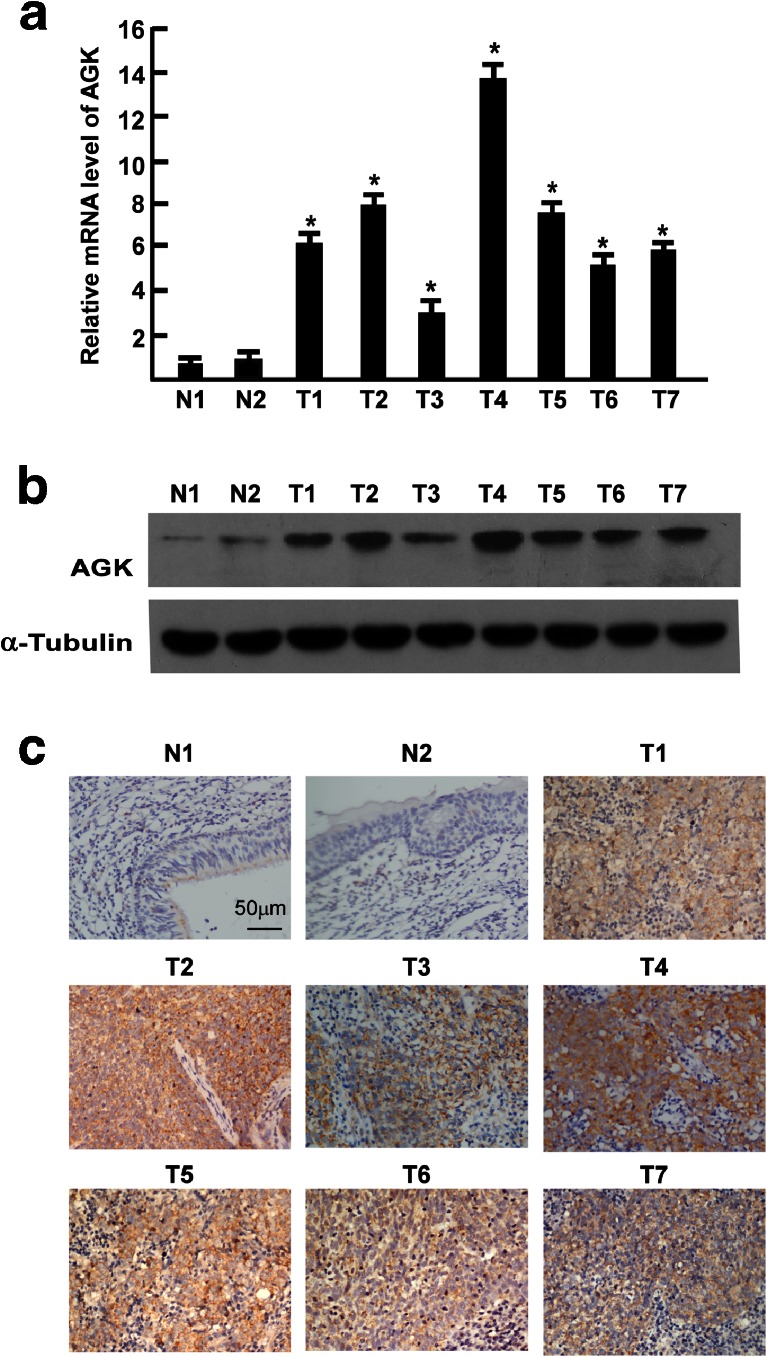
Overexpression of AGK mRNA and protein in NPC. **a** AGK mRNA expression in two paired tumor samples (T1–T2) and the adjacent noncancerous tissues (N1–2) from the same patients, and five additional tumor samples (T3–7) was quantified by q-PCR and normalized to GAPDH. *Error bars* are standard deviation of the mean (SD) for three experiments performed in parallel. *P < 0.05. **b** Representative Western blotting analyses of AGK protein expression in the two paired tumor samples and adjacent noncancerous tissues from the same patients and five additional tumor samples; α-tubulin was used as the loading control. **c** Immunohistochemical analysis of AGK protein expression in the two paired tumor samples and adjacent noncancerous tissues from the same patients and five additional tumor samples

To investigate whether AGK is overexpressed in human NPC, two paired tumor samples and the adjacent noncancerous tissues from the same patients and five additional tumor samples from other patients were subjected to quantitative real-time PCR and Western blotting analyses. As shown in Fig. 2a, AGK mRNA was significantly upregulated in all of the clinical NPC samples compared to the normal nasopharyngeal tissues. The fold increases, as indicated by the tumor/normal mRNA ratio, ranged from 3.4- to 15.6-fold. Consistent with the mRNA levels, AGK protein expression was also upregulated in the seven NPC tissues tested compared to the two normal nasopharyngeal tissues (Fig. 2b). Taken together, these results demonstrate that AGK is upregulated in NPC cell lines and tissues.

### Elevated AGK expression in NPCs associates with advanced clinicopathological features of the patients

**Fig. 3:**
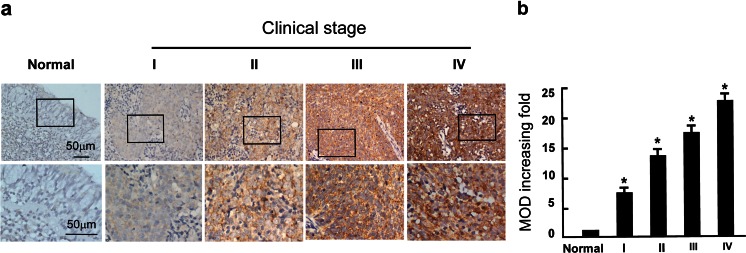
Expression of AGK in different clinical stages of NPC. **a** Representative IHC images of AGK expression in normal nasopharyngeal tissues and NPC tissues of different clinical stages. **b** Statistical analyses of the average MOD of AGK staining between normal human nasopharyngeal tissues and NPC specimens of different clinical stages. *P < 0.05

We further analyzed the correlation between AGK expression and the clinicopathological features of NPC. As shown in Fig. 2c, AGK was strong cytoplasmic staining in the NPC tissue, while barely detectable in the normal epithelial cells. Moreover, *χ*2 test showed that there was no significant association between AGK protein expression and patient age or gender. However, AGK was significantly associated with clinical stage (*P* < 0.001), T classification (*P* = 0.012), N classification (*P* < 0.001) M classification (*P* = 0.006), and histological differentiation (*P* < 0.001). Furthermore, IHC staining showed that AGK expression in the NPC increased with increasing clinical stage (Fig. 3a). Quantitative analysis also revealed that the average mean absorbance of AGK staining in stage I–IV tumors was statistically significantly higher than in the normal nasopharyngeal tissues. In addition, the mean optical density (MOD) values of AGK staining significantly increased with progression of tumor stage from I to IV (*P* < 0.05, Fig. 3b). Spearman’s rank correlation analysis suggested that high AGK expression correlated positively with clinical stage (*r* = 0.410, *P* < 0.001), T classification (*r* = 0.290, *P* = 0.002), N classification (*r* = 0.517, *P* < 0.001), M classification (*r* = 0.257, *P* = 0.006), and histological differentiation (*r* = 0.414, *P* < 0.001, Table 2). However, no significant correlation was observed between AGK expression and any other clinical feature including age or gender. Taken together, these data indicate that elevated AGK expression is associated with disease progression in NPC.

**Table 2:** Spearman correlation analysis between AGK and clinical pathologic factors

Variables	Spearman correlation	*P* value
Clinical stage (I vs II vs III vs IV)	0.410	<0.001
T classification (T1 vs 2 vs 3 vs 4)	0.290	0.002
N classification (N1 vs 2 vs 3 vs 4)	0.571	<0.001
M classification (M0 vs 1)	0.257	0.006
Histological differentiation (U vs D)	0.414	<0.001
Lymph node metastasis (yes vs no)	0.362	<0.001
Vital status (live vs die)	0.425	<0.001
Gender (M vs F)	0.016	0.869
Age, years (≥45 vs <45)	0.094	0.319

*M* male, *F* female

### Elevated AGK expression in NPC associates with poorer patient survival and prognosis

**Fig. 4:**
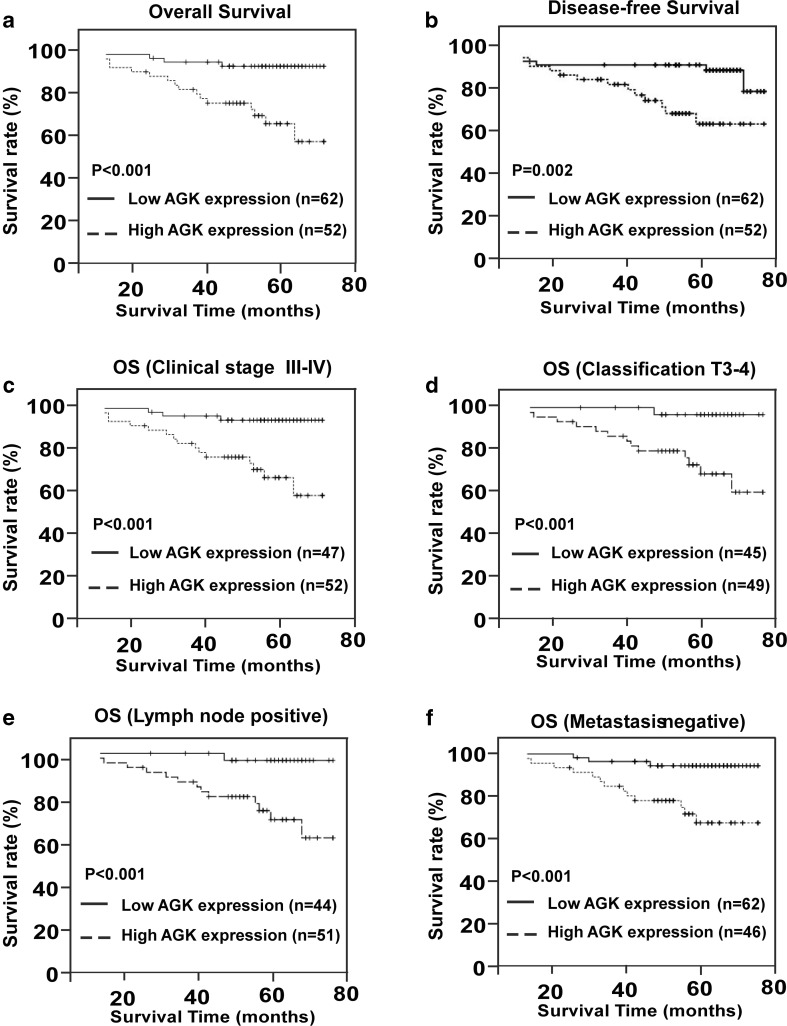
Five-year overall survival (**a**) and 5-year disease-free survival (**b**) for 114 NPC patients and 5-year overall survival for the subgroups of patients with stage III–IV disease (**c**), T3–T4 grade tumors (**d**), lymph node metastasis (**e**), and patients without distant metastasis (**f**). *P* values were calculated using the logrank test

Kaplan-Meier survival analysis revealed significant associations between high AGK protein expression and poorer 5-year OS and DFS in NPC patients (*P* < 0.001 and *P* = 0.002; Fig. 4a, b). The cumulative 5-year OS rates and DFS rates for patients with high levels of AGK expression were 67.3 and 76.9 %, respectively, in comparison to 98.4 and 93.5 %, respectively, for patients with low or no AGK expression. Median follow-up times for patients with low AGK expression was 58 months compared to 38 months for patients with high AGK expression.

Multivariate Cox regression demonstrated that the expression of AGK, clinical stage, and M classification were independent prognostic factors for poorer OS (*P* = 0.041, *P* = 0.040, *P* = 0.010, respectively; Table 3). Moreover, the prognostic value of AGK was analyzed when the patients were stratified according to tumor stage and T, N, and M classification. High AGK expression was a significant prognostic factor for poorer OS in patients with late-stage disease (stage III–IV, *P* < 0.001; Fig. 4c), patients with a pT3–4 classification (*P* < 0.001, Fig. 4d), patients with neck lymph node metastasis (*P* < 0.001, Fig. 4e), and patients without distant metastasis (*P* < 0.001, Fig. 4f). However, no statistically significant associations were observed between AGK and OS in the early stage disease (stage I–II), neck node negative, or distant metastasis-positive subgroups, which may reflect the limited number of patients in these subsets. Taken together, these results indicate that AGK could be a useful prognostic factor in NPC.

**Table 3:** Univariate and multivariate Cox regression analysis of the association between various prognostic features and overall survival in nasopharyngeal cancer

Variable	Univariate		Multivariate		
	*P*	Regression coefficient (SE)	*P*	Relative risk	95 % CI
AGK expression (high vs low)	0.001	3.298 (1.031)	0.041	8.882	1.097–7.929
T stage (T1 vs 2 vs 3 vs 4)	0.010	1.031 (0.401)	0.510	0.633	0.162–2.471
N stage (N1 vs 2 vs 3 vs 4)	<0.001	1.066 (0.281)	0.300	1.447	0.720–2.909
M stage (M0 vs 1)	<0.001	2.414 (0.532)	0.010	4.616	1.439–4.878
Clinical stage (I vs II vs III vs IV)	0.001	2.014 (0.606)	0.040	6.163	1.083–5.090

### Elevated AGK expression in NPCs associates with lymph node metastasis

NPC has the highest preponderance for regional lymph node metastasis among head and neck squamous cell carcinomas. Therefore, we evaluated the association between the expression of AGK and lymph node metastasis. In our study, there was significant difference between the immunohistochemical status of AGK protein expression in patients with lymph node metastasis and patients without lymph node metastasis (*P* < 0.001; chi-squared test). In the subgroup of patients with lymph node metastasis, patients with higher levels of AGK expression had a shorter survival time compared to those with lower AGK expression. Furthermore, multivariate logistic regression analysis showed that high AGK protein expression (*P* = 0.032), histological differentiation (*P* = 0.026), and T classification (*P* = 0.015) were significantly associated with lymph node metastasis in NPC; however, age and gender showed no association with lymph node metastasis (Table 4). Taken together, these results suggest that AGK could play an important role in lymph node metastasis in NPC.

**Table 4:** Multivariate logistic regression analysis of factors associated with lymph node metastasis in NPC

Parameters	*B*	S.E.	Wald	*P*	Exp (B)	95.0 % CI for Exp (B)	
						Lower	Upper
AGK expression (high vs low)	2.362	1.102	4.594	0.032	5.613	1.224	92.034
Histological differentiation (U vs D)	1.462	0.655	4.986	0.026	4.315	1.196	15.573
T stage (T1–2 vs T3–4)	1.697	0.697	5.927	0.015	5.458	1.392	21.400
Age, years (≥45 vs <45)	−1.278	0.680	3.528	0.060	0.279	0.073	1.057
Gender (M vs F)	−0.043	0.684	0.004	0.950	0.958	0.251	3.660

## Discussion

AGK has been suggested to promote tumorigenesis in various cancers, including ESCC, hepatocellular cancer, and breast cancer [12, 13, 18]. MTT and colony formation assays showed that overexpression of AGK increased the proliferation of breast cancer cells, whereas silencing AGK drastically reduced cell proliferation [12]. In xenograft experiments, ESCC cells overexpressing AGK showed an increased growth rate and tumorigenic capacity [13]. Data from a recent mouse model showed that the tumors formed by AGK-transduced hepatocellular carcinoma (HCC) cells grew more rapidly and were larger in size, while the tumors formed by AGK-silenced cells were smaller in both size and weight, compared to the tumors formed by control cells [18]. Taken together, these results confirmed that overexpression of AGK could contribute to the proliferation of tumor cells, indicating that AGK is involved in the progression of cancer. Consistent with the above research, AGK was reported to be associated with the development and progression of several types of solid carcinoma [8, 14–18]. In the present study, we observed significant associations between AGK expression and the clinicopathologic characteristics of NPC, including clinical stage, TNM classification, and histological differentiation. Furthermore, survival analyses showed that patients with higher levels of AGK expression had shorter survival time compared to those with lower AGK expression. Additionally, Cox regression analysis further confirmed that AGK may be an independent prognostic factor for poor overall survival in patients with NPC. Collectively, these findings indicate that AGK may contribute to the development and progression of NPC.

Invasion and metastasis are the basic biological characteristics related to recurrence and also affect the survival of patients with NPC [19–21]. Due to the well-developed network of lymph nodes in the nasopharynx, NPC has the highest preponderance for regional lymph node metastasis among head and neck squamous cell carcinomas [22]. In this study, we observed significantly higher AGK protein expression in patients with NPC with lymph node metastasis compared to those without lymph node metastasis. Furthermore, in the subgroup of patients with lymph node metastasis, patients with higher levels of AGK expression had a shorter survival time compared to those with lower AGK expression. Additionally, high expression of AGK was an independent prognostic factor for lymph node metastasis in NPC. These results strongly support the hypothesis that high levels of AGK expression play a critical role in promoting lymph node metastasis in NPC. Previous studies have demonstrated important associations between metastasis and overexpression of vascular endothelial growth factor (VEGF)/epidermal growth factor (EGF) in a variety of solid carcinomas, including NPC [23, 24]. VEGF-induced lymphangiogenesis and EGF-induced angiogenesis can enhance the development of lymphatic and vessels within and close to tumors and thereby promote the spread of tumor cells to regional lymph nodes [25, 26]. Moreover, overexpression of AGK transactivates the epidermal growth factor receptor (EGFR) and increases prostate cancer cell migration in vitro [8]. Additionally, the increased tumorigenicity observed in response to AGK-mediated downregulation of FOXO1 in breast cancer may be due to aberrant activation of AKT [15], which is a major downstream effector of the EGFR [27, 28]. Genome wide survey of multiple oncogene amplifications found that EGFR were involved in the development of NPC [29]. Recently, a report suggested that ectopic expression of AGK can enhance the expression of VEGF and promote angiogenesis in HCC in vitro [18]. On the basis of this evidence, we assume that high levels of AGK may promote lymph node metastasis in NPC via VEGF or/and EGFR. However, further investigation is required to confirm this hypothesis.

In summary, this is the first study to highlight the clinical significance of AGK in NPC. High AGK expression was associated with poorer survival and lymph node metastasis in patients with NPC. Comprehensive analysis of the molecular mechanisms underlying the role of AGK in the development and progression of NPC is warranted.

## Conclusions

This study demonstrates that overexpression of AGK correlates with disease progression in NPC, indicating that AGK has value as a novel prognostic biomarker. Further exploration of the exact function of AGK during the progression of NPC is required.
